# Understanding the Dynamic Convergence Phenomenon from the Perspective of Diversity and Persistence: A Cross-Sector Comparative Analysis between the United States and South Korea

**DOI:** 10.1371/journal.pone.0159249

**Published:** 2016-07-14

**Authors:** We Shim, Oh-jin Kwon, Yeong-ho Moon, Keun-hwan Kim

**Affiliations:** 1 S&T Information Science, Korea University of Science and Technology (UST), Seoul, Korea; 2 Department of Scientometric Research, Korea Institute of Science and Technology Information (KISTI), Seoul, Korea; 3 Future Information Research Center, Korea Institute of Science and Technology Information (KISTI), Seoul, Korea; 4 Korea Institute of Science and Technology Information (KISTI), Seoul, Korea; 5 Department of Enterprise Innovation Strategy, Korea Institute of Science and Technology Information (KISTI), Seoul, Korea; Utrecht University, NETHERLANDS

## Abstract

This study was designed to improve the explanation for the behavior of the phenomenon of technology convergence. The concepts and measurements of diversity and persistence, as inherent attributes of the phenomenon, were elaborated by reviewing different theories. Diversity was examined by analyzing the degree of capability to absorb heterogeneous technologies, while persistence was investigated by analyzing the degree of continuity in the usage of cumulated technologies. With these two dimensions, an analytic framework was proposed to compare the differences and dynamic patterns of convergence competence by countries at the technology sector level. Three major technology sectors in the United States and South Korea, namely, information and communication technology, biotechnology, and nanotechnology, were explored to explicitly illustrate the differences in technology convergence competence. The results show that although Korea has narrowed the differences of capabilities for technology convergence compared to the US, Korea not only has to continuously pursue the improvement of specialization for all three sectors, but also has to encourage the exploitation of different technology fields. The suggested framework and indicators allow for monitoring of the dynamic patterns of a technology sector and identifying the sources of the gaps. Thus, the framework and indicators are able to ensure the purpose of government innovation policy and to provide strategic directions for redistributing the proper combination of sources to accomplish technology convergence.

## Introduction

The phenomenon of convergence has been interpreted as a confluence process through merging hitherto separated industries, generally triggered by new science discoveries and technological developments, which remove entry barriers across the industries and set in motion evolutionary development with a broader impact on global economics [1]. The consequential outcomes enable an increase in opportunities in a wide variety of industries. Thus, the sum of technological trajectories may outperform that of their parts [2]. Since the 1990s, economically developed countries have set up national plans to improve the capabilities of technology convergence, enhancing their economic growth [3]. The growing importance of technology convergence has been reflected in the extant literature that addresses the benefits and impact of convergence [2, 4–5]. Despite this increasing interest, prior studies focused on the technological developments [6–7], management practice [1, 5, 8], and new business opportunities [9–11] that stem from newly converging industries. These studies appear to have been analyzed with little attention to an in-depth examination of the inherent nature of technology convergence. Consequently, they have failed to create a broader consensus on the appropriate methodology. Hacklin, Marxt, and Fahrni [2] argued that one of the biggest obstacles to achieving such consensus might be the lack of understanding, per se, of the convergence phenomenon. Without seeing through the nature of the phenomenon, developing measurements would be insignificant for promoting cross-fertilization of knowledge in multiple technology fields. Thus, it is necessary to build measurements based on reflecting the inherent nature of technological convergence. Addressing such a requirement can provide the primary basis for comparing the differences in the degree of technological convergence across countries, enabling identification of the reasons for the gap in technological convergence and suggesting appropriate strategies to bridge the gap.

To bring these issues in focus, the objectives of this study are:

1. to study the mechanism underlying the phenomenon of technology convergence and to suggest valid concepts that may describe the mechanism thoroughly;
2. to suggest indicators that reflect the concepts of technological convergence and to investigate technology sectors in which the convergence phenomenon have occurred at the country level; and
3. to assess usefulness and limits by describing the dynamics of a country’s relative differences in technology sectors.

The first step is to review the extant theories carefully, rather than taking at face value the interpretation of convergence, as described above. Thus, we examine the phenomenon of convergence through various theories to build key concepts that may pertinently construct the phenomenon of technology convergence. To begin with, historians of technology have tried to explain convergence logically using rational illustrations. Moreover, some seminal studies [12–14] have provided insight to understand the concept of technology convergence, and regarded it as a process that occurs largely to solve problems with solutions that are beyond the scope of a specific technology field. Such continuous processes produce unexpected innovations, which then inevitably influence other technology fields. Thomson [14] stressed that greater innovation is always created by self-usage, and that evolutionary problem-solving activities, sustained by a dynamic competitive selection mechanism, stand as the basis of the learning process of interactions with firms in different areas. This viewpoint is based on a number of doctrines, including the resource-based view [1, 5, 15–17] and evolutionary theory [18–22].

Within resource-based theory, many scholars have focused on understanding the resources that differentiate the competitive advantages among firms. Technology convergence was found to be triggered by a combination of various factors, including deregulation, product bundling, and integrative technologies [17]. These factors force firms to invent new products and services in their originating industry, gradually expanding to share increasingly comparable technical- and market-based characteristics with different industries [5]. Thus, the competitive structure of a given industry and the nature of a firm’s core competence are fundamentally changed. Firms then concentrate on establishing accumulated learning experiences by developing diverse products and services as well as cultivating multiple sets of dynamic routines that generate competencies across industries [15–16].

Research on evolutionary economics describes how the evolution of firm and industry structure has been transformed through a process of technological advances, competing through a variety of new alternatives, and selecting dominant technologies that achieve cumulative improvements in the market environment [19]. At the heart of the evolutionary process is the need for a firm to provide more market-matched products and services using heterogeneous technologies than its rivals, and then, to be favored by customers. Where firms or industries constantly use standardized patterns of action, they can easily achieve this goal because such routines become the foundation for their outperformance of rivals [22].

From reviewing the aforementioned theories, we conclude that the phenomenon of technology convergence has been transformed by the processes of (1) integrating heterogeneous technologies and (2) enhancing the consistent utilization of aggregated technologies. Such a notion is consistent with that of Lemola [23], who argued that “convergence is an integral part of an organizational learning process oriented, on one hand, to the exploration of new possibilities and, on the other hand, to the exploitation of old certainties” (p. 1482). Thus, we argue that the convergence phenomenon occurs due to the mechanism that performs the interactive process of two inherent concepts.

Once we establish the important concepts responsible for the convergence phenomenon, we can move to the second step to develop valid measurements that reflect the meaning of the two concepts. Rafols and Meyer [24] suggested a framework to capture the process of knowledge integration using two measurements: diversity and coherence. The abovementioned concepts and measurements, which reflect the inherent characteristics of interdisciplinarity, are defined and proposed in Table 1.

**Table 1:** Definition, Inherent Characteristics, and Indexes of Interdisciplinarity.

**Definition**	Interdisciplinarity: The process of integrating different bodies of knowledge rather than being restricted by the boundary per se [24–26].	
**Inherent characteristics**	Diversity: An attribute of any system whose elements may be apportioned into categories [27].	Coherence: The extent to which a system’s elements are consistently articulated and form a meaningful constellation [28].
**Index**	Rao–Stirling: $D=\sum_{ij}d_{ij}^{\alpha }{\left(p_{i}p_{j}\right)}^{\beta }$	Mean linkage strength (the mean degree centrality) and Mean path length (the mean of closeness centrality)

Although their study [24] did not establish definitions based on comprehending the inherent characteristics of interdisciplinarity by reviewing theoretical literature, it proposed grounds for a conceptually well-integrated framework to understand the complex process, by focusing on bibliometric studies on interdisciplinarity. The distinctive characteristics of interdisciplinarity are in accordance with those of technology convergence and, therefore, it is reasonable to adopt indexes for the present study, in order to grasp the convergence phenomenon.

After publishing their studies, many researchers have paid attention to the topic. In particular, developing a new measurement for coherence was called for to reflect the very nature of the concept, and Rafols, Leydesdorff, O’Hare, Nightingale, and Stirling [28], as well as Soós and Kampis [29], responded to this request. Table 2 presents a summary of a comparison of these studies. Like these researchers, we agree that the term “diversity” and its well-known indicator—the Rao-Stirling index—are fit for the first conception of this study. Then, we need to examine the indexes closely for coherence, as suggested by these scholars, to decide whether to adopt them for the second concept in the present study.

**Table 2:** Comparison among Studies Concerning Coherence Indicators.

	Rafols and Meyer [24]	Rafols, Leydesdorff, O’Hare, Nightingale, and Stirling [28]	Soós and Kampis [29]
Dimensions	Diversity and coherence	Diversity, coherence, and intermediation	Diversity and field/disciplinary coherence
Indicators for coherence	Coherence: *S* = Mean linkage strength and *L* = Mean path length	Coherence: $\frac{\sum_{i,j}p_{ij}d_{ij}}{\sum_{i,j}p_{i}p_{j}d_{ij}}$	Disciplinary Coherence: $\sum_{i=1,j=1}^{n}p_{ij}d_{ij}c_{ij}$

First, we investigated Rafols and Meyer’s [24] study, which attempted to determine the various degrees of knowledge integration, building upon the notions of diversity and coherence. In detail, network coherence was operationalized by choosing a similarity metric between network elements (articles) to measure the strength of their linkages as well as their distribution. The degree of similarity was calculated via bibliographic couplings between the co-occurrences of the references, which were regarded as network centralities. Mean linkage strength *S* and mean path length *L* in the network were computed to measure the means of both degree and closeness centrality.

Moreover, Rafols, Leydesdorff, O’Hare, Nightingale, and Stirling [28] argued that coherence is expressed in the form of the expected value (adopted from Rao–Stirling diversity) of the denominator and observed value of the numerator (joint research actually occurred in two units within the publication). The observed/expected ratio indicated whether the unit under exploration was linking distant categories within its publication portfolio.

Soós and Kampis [29] proposed the term “interdisciplinary network,” or “*i*-network.” In their model, field coherence and disciplinary coherence were introduced to measure the degree of interdisciplinarity. Disciplinary coherence was adapted by modifying the numerator of field coherence, as noted in Rafols, Leydesdorff, O’Hare, Nightingale, and Stirling [28]. This modified measurement is weighted by the actual distances (*d*_*ij*_) with variable (*c*_*ij*_). The variable functions as a filter because it has the value of 0 if discipline (*i*) is identical to discipline (*j*); otherwise, the value is 1.

The abovementioned studies share a common thread: the suggested indexes for coherence do not reflect a continuous dynamic process of knowledge integration, but instead examine the structure of integration. As mentioned earlier, there is a conceptual robust similarity between Rafols and Meyer’s [24] study and the present study. However, the indexes for coherence discussed above are not adequate to measure the second conception suggested in this article. Therefore, we recognize the need to suggest an appropriate index that reflects the notion of the continuous exercises of the aggregated technologies. In addition, the reconsideration of the employment of the term “coherence” is required, due to its deficiency as a means of valid measurement.

The second concept in this study can be described simply as the accumulation of technological capabilities. Seminal economic historians [30–34] have already explained that it stems from deliberate internal endeavors as well as cumulative learning processes. Moreover, Cefis and Orsenigo [35] stated that the cumulativeness plays a fundamental role in generating new knowledge and is determined by the degree of continuity in innovative activities over time. In other words, knowledge development based on established practices is dependent on the existence of persistence. Two studies [36–37] provided evidence that strong persistence is associated with the greatest innovators, while Brusoni and Geuna [38] emphasized that persistence, as the stability of specialization patterns in specific technological fields, plays a dominant role in determining national competitiveness. In consideration of the bodies of literature discussed above, we consider that persistence is suitable to reflect the second concept in this study. Concerning its measurement, Pearson’s correlation coefficient, as suggested by Pavitt [39] as well as Brusoni and Geuna [38], is deliberated to verify the stability of the persistence patterns for the period considered. However, Ahlgren, Jarneving, and Rousseau [40] claimed that many zeros distort the values of Pearson’s correlation coefficient, thereby obfuscating the precise interpretation of the results obtained using this method. In order to prevent such misinterpretation, we adopt cosine similarity, rather than Pearson’s correlation coefficient, because it takes into account only the similarities in the non-zero dimensions.

**Fig 1:**
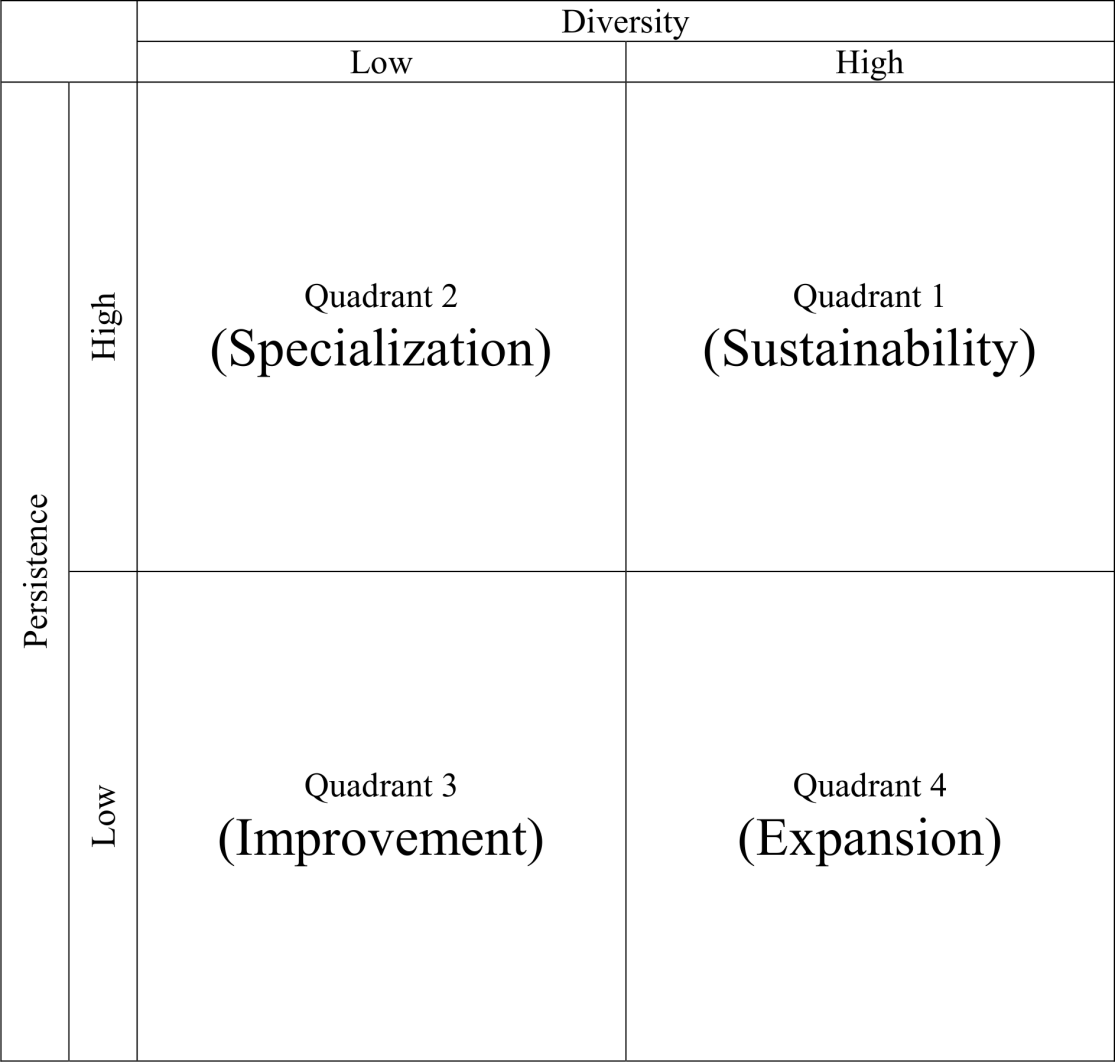
Analytical Framework for Understanding Technology Convergence.

In summary, we researched the mechanism for the phenomenon of technology convergence through three different theoretical lenses, thereby deducing two attribute-based phenomenon concepts—diversity, which is the degree of capability to absorb heterogeneous technologies, and persistence, which is the continuity in the usage of cumulated technology. Moreover, the appropriate methodology is suggested for each concept. Based on a sound conceptualization of two dimensions, the phenomenon of technology convergence can be explained as continuous improvement processes by interaction of the activities of diversity and persistence, as shown in Table 3, and we can propose a framework to aid understanding of the meaningful differences among phenomena at country level, as shown in Fig 1.

**Table 3:** Definition, Inherent Characteristics, and Methods of Technology Convergence.

**Definition**	The phenomenon of technology convergence is a process of integrating heterogeneous technologies and then enhancing the consistent utilization of aggregated technologies	
**Inherent characteristics**	Diversity: the degree of capability to absorb different technologies	Persistence: the degree of continuity in the usage of cumulated technology
**Index**	Rao-Stirling index	Cosine similarity

This framework is capable of combining the analysis on the degree of innovation activities from diverse fields with that on the degree of innovation activities in possessed knowledge over time. It should shed light on the dynamic characteristics of the technology convergence of the subject (e.g., nations, companies, or individuals) of study in specific sectors and, thus, help to show the relative differences in capabilities of technology convergence and to clarify the sources of such a gap of the continuously changing convergence phenomenon.

With regard to any specific sector, the subject can be tracked to the dynamic pattern of technology convergence in the four quadrants of its matrix. By investigating two large Japanese firms, Suzuki and Kodama [41] demonstrated that the existence of both the diversity and persistence of technology contributes to sales growth. Thus, we can assume that the higher is a nation’s level of capabilities in technological convergence, the higher economic performance it creates. As such, a subject in Quadrant 1 is characterized by both high diversity and persistence capabilities of technologies, so that it can sustain its economic developments in wide-range industries (sustainability). On the contrary, a subject in Quadrant 3 is represented by both low diversity and persistence. It is obvious that it will try to improve technological competences to increase economic growth and prosperity (improvement). At this point, the subject may follow the route toward Quadrant 1 via Quadrant 2 rather than via Quadrant 4. A subject in Quadrant 2, with low diversity and high persistence, possesses a special capability in a specific field, while persistently using a body of existing knowledge over a period of time (specialization). By contrast, a subject in Quadrant 4, with high diversity and low persistence, may have a strategic goal to enter a wide range of industries as fast as possible (expansion). Perez and Soete [42] explained that the higher is the level of relevant scientific and technical knowledge already possessed by the firm, the higher is its capacity to absorb new knowledge. Thus, a subject soon encounters the limitation of sustainable growth because it has to pay a high cost to gain the related knowledge. Therefore, it is expected that the subject in Quadrant 3 may take a route toward Quadrant 2.

## Materials and Methods

We operationalize our framework in the case of the information and communication technology (ICT), biotechnology (BT), and nanotechnology (NT) sectors of the United States (US) and South Korea (Korea). The main reason we choose these three technology sectors is that they are under the global spotlight as the growth engines of nations [43–48]. Moreover, the US plays an important role in leading these technology sectors and has contributed considerably to the birth of these sectors compared to other countries [49–50]. On the other hand, Korea has set an example in its remarkable improvement in technological capabilities since the 1990s. Specifically, it has stood out preferentially in the global ICT sector since the early 2000s [51], and has begun to attempt the same success in BT and NT [52–53].

By illustrating the differences between the technology sectors in these two countries, which show discrepancies in their technology convergence capacity, we investigate the relative technological positions of these two countries as well as their strengths and weaknesses in the technology sectors, thereby providing specific policy implications.

### Materials

Patent documents are an ample source for technical knowledge about technology progress and innovative activity, and many studies have utilized patent data to measure technology convergence [54]. However, we are aware of the limitations of patents. The propensity for patents to vary substantially across countries depends on a variety of factors, such as the intensity of commercial relations and the effectiveness of the protection. Therefore, many studies have used patents granted at the US Patent Trademark Office (USPTO) with the assumption that the US market is the largest and most technologically developed in the world [55]. In addition, firms sometimes choose alternative methods to protect their innovations. Thus, an unknown number of inventions are not patentable [56]. However, Archibugi [57] and Acs, Anselin, and Varga [58] argued that the measure of patented inventions provides a reliable representation for innovative activity. Moreover, there are various advantages of the use of a patent. Patent data are available for a very long time series, and thus, they allow researchers to investigate technology dynamics from a long-term perspective [59]. In addition, patent documents are classified in accordance with the International Patent Classification (IPC) system, which represents a searchable collection of patents grouped together according to similarly claimed subject matter. Thus, many researchers have employed the system to analyze the technology convergence phenomenon [60–64]. In particular, IPC codes have been recognized as technological fields and utilized to measure the degree of technological positions [65]. The Organisation for Economic Co-operation and Development (OECD) has designated specific IPC codes for the ICT, BT, and NT sectors, as shown in Table 4 [50, 66].

**Table 4:** ICT, BT, and NT Sectors by IPC Codes.

Sector		IPC Code
**Information and communication technology (ICT)**	**Telecommunications (TEL)**	G01S, G08C, G09C, H01P, H01Q, H03B, H03C, H03D, H03H, H03M, H04B, H04J, H04K, H04L, H04M, H04Q, H01S
	**Consumer electronics (CE)**	G11B, H03F, H03G, H03J, H04H, H04N, HO4R, HO4S
	**Computers and office machinery (COM)**	B07C, B41J, B41K, G02F, G03G, G05F, G09G, G10L, G11C, H03K, H03L
**Biotechnology (BT)**		A01H, A61K, C02F, C07G, C07K, C12M, C12N,C12P, C12Q, G01N
Nanotechnology (NT)*		B01J, B81B, B82B, C01B, C01G, C03B, C03C, C23C

Source: OECD [50, 66]

*This study selected only 4-digit IPC codes among nanomaterials suggested by the OECD to be nanotechnology [66].

**Fig 2:**
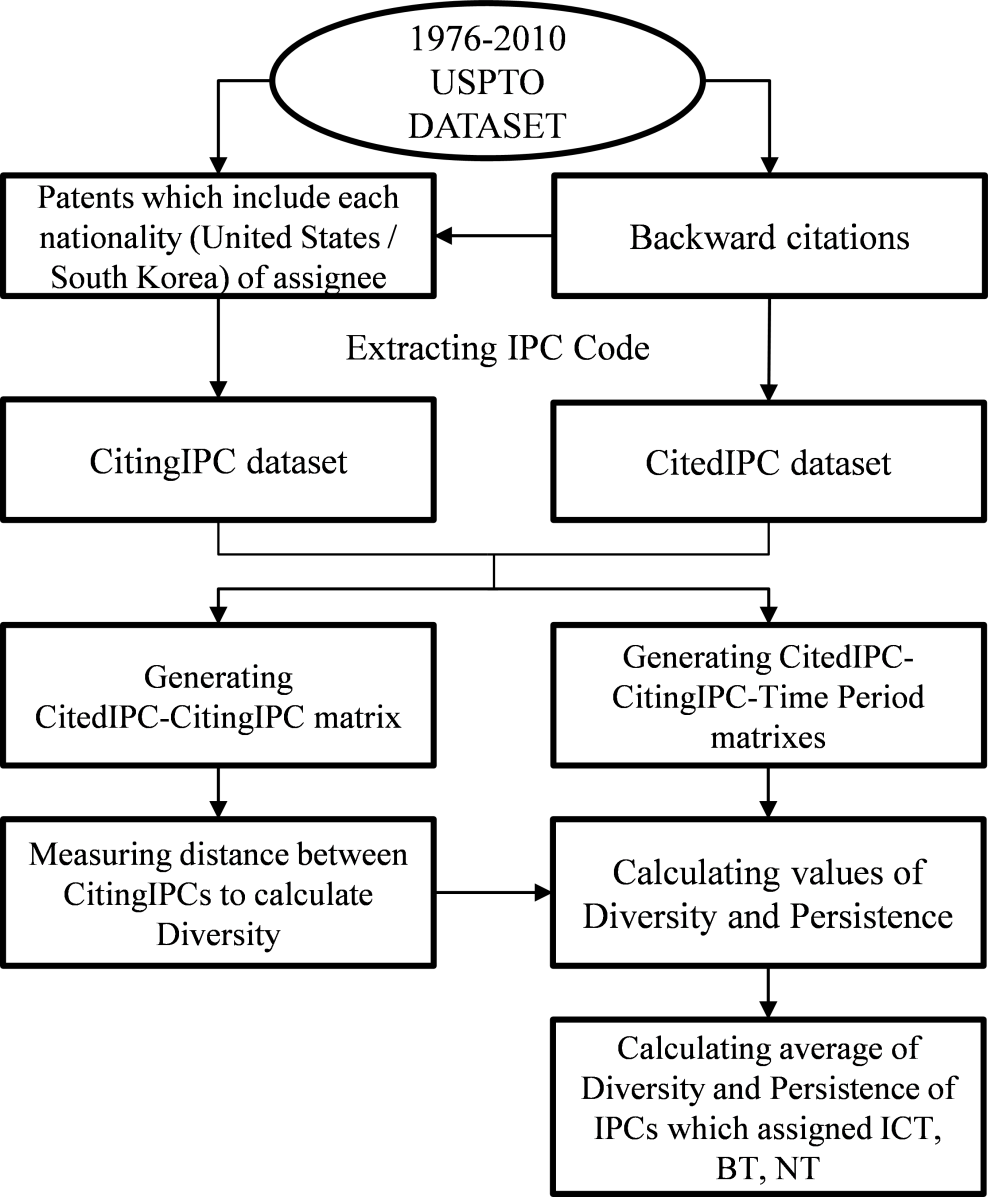
Diagram of the Data Analysis Process.

In this study, on the basis of patent data from the USPTO, we use specific IPC codes for the ICT, BT, and NT sectors designated by the OECD [50, 66], as shown in Table 3, to track convergence patterns among these technology sectors for two decades (1991–2010). The choice of this time period is appropriate because it may cover the beginning and progression of technological convergence among selected sectors. A general explanatory diagram of our data analysis process is shown in Fig 2.

First, the cited–citing patent dataset by year is established based on patents granted by the USPTO. The basic information of this citation dataset from 1991 to 2010 is shown in Table 5.

**Table 5:** Basic Information from Patent Citation Dataset.

Year	# of Citing Patents	# of Citing Patents (US)	# of Citing Patents (KR)	# of Cited Patents	# of Patents Cited by Citing Patents (US)	# of Patents Cited by Citing Patents (KR)
1991	88,060	37,547	303	167,667	109,710	1,146
1992	89,932	38,877	425	134,702	95,012	1,502
1993	91,735	40,679	637	120,697	92,754	2,350
1994	95,847	43,447	833	115,027	93,609	3,257
1995	96,029	43,278	1,046	108,702	89,529	3,998
**1991–1995**	**461,603**	**203,828**	**3,244**	**646,795**	**480,614**	**12,253**
1996	100,184	46,000	1,279	106,169	89,372	4,984
1997	105,737	48,741	1,757	104,808	88,993	6,692
1998	139,491	63,844	3,028	130,524	111,744	12,227
1999	145,372	66,959	3,331	136,447	115,448	13,706
2000	150,234	68,852	3,082	140,888	119,061	13,420
**1996–2000**	**641,018**	**294,396**	**12,477**	**618,836**	**524,618**	**51,029**
2001	159,048	72,756	3,259	150,642	127,198	14,449
2002	160,666	73,135	3,469	152,628	128,458	14,293
2003	163,236	74,784	3,598	150,876	130,970	13,997
2004	158,677	72,585	4,139	141,104	122,884	16,098
2005	139,019	64,733	4,024	121,006	107,239	15,171
**2001–2005**	**780,646**	**357,993**	**18,489**	**716,256**	**616,749**	**74,008**
2006	167,711	78,318	5,505	140,641	124,000	20,667
2007	151,420	69,930	5,918	122,761	105,629	19,683
2008	151,496	68,969	7,107	122,559	102,286	22,999
2009	158,935	73,171	8,033	126,877	106,584	25,064
2010	206,664	94,955	10,409	155,940	131,822	29,228
**2006–2010**	**836,226**	**385,343**	**36,972**	**668,778**	**570,321**	**117,641**
**Total**	**2,719,493**	**1,241,560**	**71,182**	**2,650,665**	**2,192,302**	**254,931**

The total number of patents that cited other patents (Citing Patents) during the period 1991–2010 was 2,719,493. Of them, the number of patents that included US nationality of assignees (Citing Patents (US)) was 1,241,560, while that which contained Korean nationality of assignees (Citing Patents (KR)) was 71,182. Furthermore, the number of patents cited by Citing Patents was 2,650,665, which included 2,192,302 by Citing Patents (US) and 254,931 by Citing Patents (KR).

Based on the constructed dataset, we extracted IPCs as shown in Table 6.

**Table 6:** An Example of Patents, Publication Year, and IPCs Mapping Table.

CitedPN	CitedPY	CitedIPC	CitingPN	CitingPY	CitingIPC
3949875	1976	B65D;A41B	4980927	1991	A41D
4008494	1977	A41D	4980927	1991	A41D
4096590	1978	A42B	4980928	1991	A41D;A42B
…	…	…	…	…	…

In Table 6, the CitedPN field shows the registration numbers of the patents cited by other patents; the CitedPY field indicates the registration year of the CitedPN, and the CitedIPC field represents the subclass of the IPCs for the CitedPN. In addition, the CitingPN field contains the registration numbers of the patents that cited other patents. The CitingPY field shows the registration year of the CitingPN, and the CitingIPC field is the subclass of IPCs of the CitingPN. From the citation dataset, the period matrixes are established in terms of subclasses (four digits) of IPC at the country level. Thereafter, diversity and persistence for each IPC for each period are calculated, and then, the mean value of diversity and persistence of the IPC set that consists of the ICT, BT, and NT sectors are calculated.

Finally, in this study, CitedIPC–CitingIPC matrix and CitedIPC–Citing-Time Period matrixes by year are constructed using the Knowledge Matrix developed by the Korea Institute of Science and Technology Information [67] and Python 2.7.

### Methods: Index for diversity and persistence

**Fig 3:**
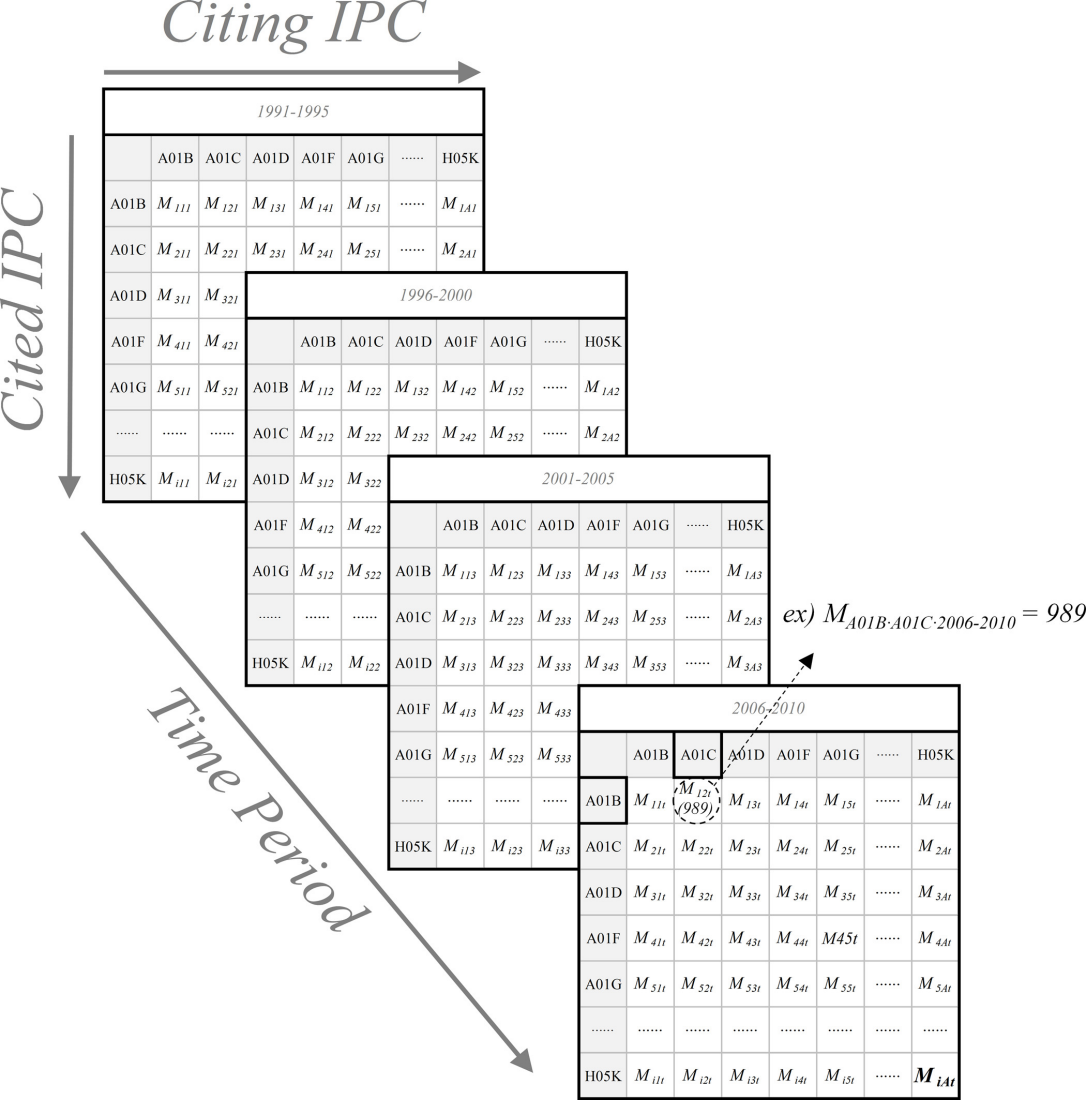
Three-Dimensional Data Structure for IPCs and Time Period. The x, y, and z axes of the three-dimensional matrix correspond to CitingIPC (Technology *A*), CitedIPC (Technology *i*), and Time Period (*t*), respectively. In each cell, *M*_*iAt*_ is the frequency with which technology *A* cites technology *i* at period *t*. For instance, if A01C (IPC Subclass) cites A01B 989 times during the period 2006−2010, then *M*_*A01B∙A01C∙(2006−2010)*_ becomes 989.

For calculating diversity and persistence indexes, it is necessary to clarify the data structure. Fig 3 shows that the three-dimensional array data structure consists of a collection of CitingIPC, CitedIPC, and Time Period.

In this study, following the previously discussed studies, we use the well-established indicator, the Rao–Stirling index, to measure diversity [27]. The diversity (*D*) index of technology *A* at the time period *t* may be computed using the following formulae:

$$\begin{array}{l} {Diversity(D)}_{At}=\sum_{i=1}^{n}\sum_{j=1}^{n}p_{iAt}∙p_{jAt}∙d_{ij}\\ \;=\sum_{i=1}^{n}\sum_{j=1}^{n}(\frac{M_{iAt}}{\sum_{i=1}^{n}M_{iAt}}∙\frac{M_{jAt}}{\sum_{j=1}^{n}M_{jAt}})∙d_{ij}\;(i\neq j) \end{array}$$ (1)

where *A* is technology that cites technology *i* (or *j*); *t* is the time period; *n* is the number of technologies (IPCs); *p*_*iAt*_ is the proportion of citations of technology *A* to technology *i* at time period *t*; *M*_*iAt*_ is the number of times technology *A* cites technology *i* at time period *t* in three-dimensional occurrence matrix *M*; and *d*_*ij*_ is the distance between technologies *i* and *j*.

For calculating distance between *i* and *j* (*d*_*ij*_), we use the cosine distance that is expressed as “*1-cosine similarity*.” A time-aggregated two-dimensional matrix (CitedIPC–CitingIPC matrix, ∑_*t* = 0_ *M*_*iAt*_) is generated to measure the cosine distance between *i* and *j*, which reflects the whole time period (1976–2010) [68]. In addition, the cosine distance is computed between vectors of CitingIPC in the CitedIPC–CitingIPC matrix. In other words, the columns of the time-aggregated matrix are vectorized. Vector ***v***_***i***_ and ***v***_***j***_ are expressed as follows:

$$v_{i}=\left[\begin{array}{c} \sum_{t=0}M_{i1t}\\ \sum_{t=0}M_{i2t}\\ .\\ .\\ .\\ \sum_{t=0}M_{iAt}\\ .\\ .\\ .\\ \sum_{t=0}M_{int} \end{array}\right],\;v_{j}=\left[\begin{array}{c} \sum_{t=0}M_{j1t}\\ \sum_{t=0}M_{j2t}\\ .\\ .\\ .\\ \sum_{t=0}M_{jAt}\\ .\\ .\\ .\\ \sum_{t=0}M_{jnt} \end{array}\right].$$

The *1-cosine similarity* between technology field *i* and *j* (*d*_*ij*_) is as follows.

$$d_{ij}=1-\frac{v_{i}∙v_{j}}{\left\|v_{i}\|\|v_{j}\right\|}=1-\frac{\sum_{q=1}^{n}{\left(v_{i}\right)}_{q}{\left(v_{j}\right)}_{q}}{\sqrt{\sum_{q=1}^{n}{{{(v}_{i})}_{q}}^{2}}\sqrt{\sum_{q=1}^{n}{{{(v}_{j})}_{q}}^{2}}}$$ (2)

where ***v***_***i***_ is a set of technologies that cite technology *i* in the whole period; and (*v*_*i*_)_*q*_ is a component of vector ***v***_***i***_.

Thus, the formula for *D* is

$${Diversity(D)}_{At}=\sum_{i=1}^{n}\sum_{j=1}^{n}\left(\frac{M_{iAt}}{\sum_{i=1}^{n}M_{iAt}}∙\frac{M_{jAt}}{\sum_{j=1}^{n}M_{jAt}}\right)\left(1-\frac{\sum_{q=1}^{n}{\left(v_{i}\right)}_{q}{\left(v_{j}\right)}_{q}}{\sqrt{\sum_{q=1}^{n}{{{(v}_{i})}_{q}}^{2}}\sqrt{\sum_{q=1}^{n}{{{(v}_{j})}_{q}}^{2}}}\right)$$ (3)

For measuring persistence, Cosine similarity is used as the degree of the utilization of the constituent technologies between a specific period and the previous period, which is computed between vectors of CitingIPC at *t-1* and *t*. Vector ***w***_***(t-1)***_ and ***w***_***t***_ are expressed as follows:

$$w_{\left(t-1\right)}=\left[\begin{array}{c} M_{1A(t-1)}\\ M_{2A(t-1)}\\ .\\ .\\ .\\ M_{iA(t-1)}\\ .\\ .\\ .\\ M_{nA(t-1)} \end{array}\right],\;w_{t}=\left[\begin{array}{c} M_{1At}\\ M_{2At}\\ .\\ .\\ .\\ M_{iAt}\\ .\\ .\\ .\\ M_{nAt} \end{array}\right].$$

The persistence of technology *A* at the time period *t* may be computed using the following formula:

$${Persistence(P)}_{At}=\frac{{w_{t}∙w}_{\left(t-1\right)}}{\left\|w_{t}\|\|w_{\left(t-1\right)}\right\|}=\frac{\sum_{q=1}^{n}{{\left(w_{t}\right)}_{q}(w_{\left(t-1\right)})}_{q}}{\sqrt{\sum_{q=1}^{n}{{{(w}_{t})}_{q}}^{2}}\sqrt{\sum_{q=1}^{n}{{{(w}_{\left(t-1\right)})}_{q}}^{2}}}\;(t\geq 2)$$ (4)

where ***w***_***t***_ is a set of technologies that is cited for technology *A* at time period *t*; *(t-1)* is the previous time period of *t*; and (*w*_*t*_)_*q*_ is a component of vector ***w***_***t***_.

**Fig 4:**
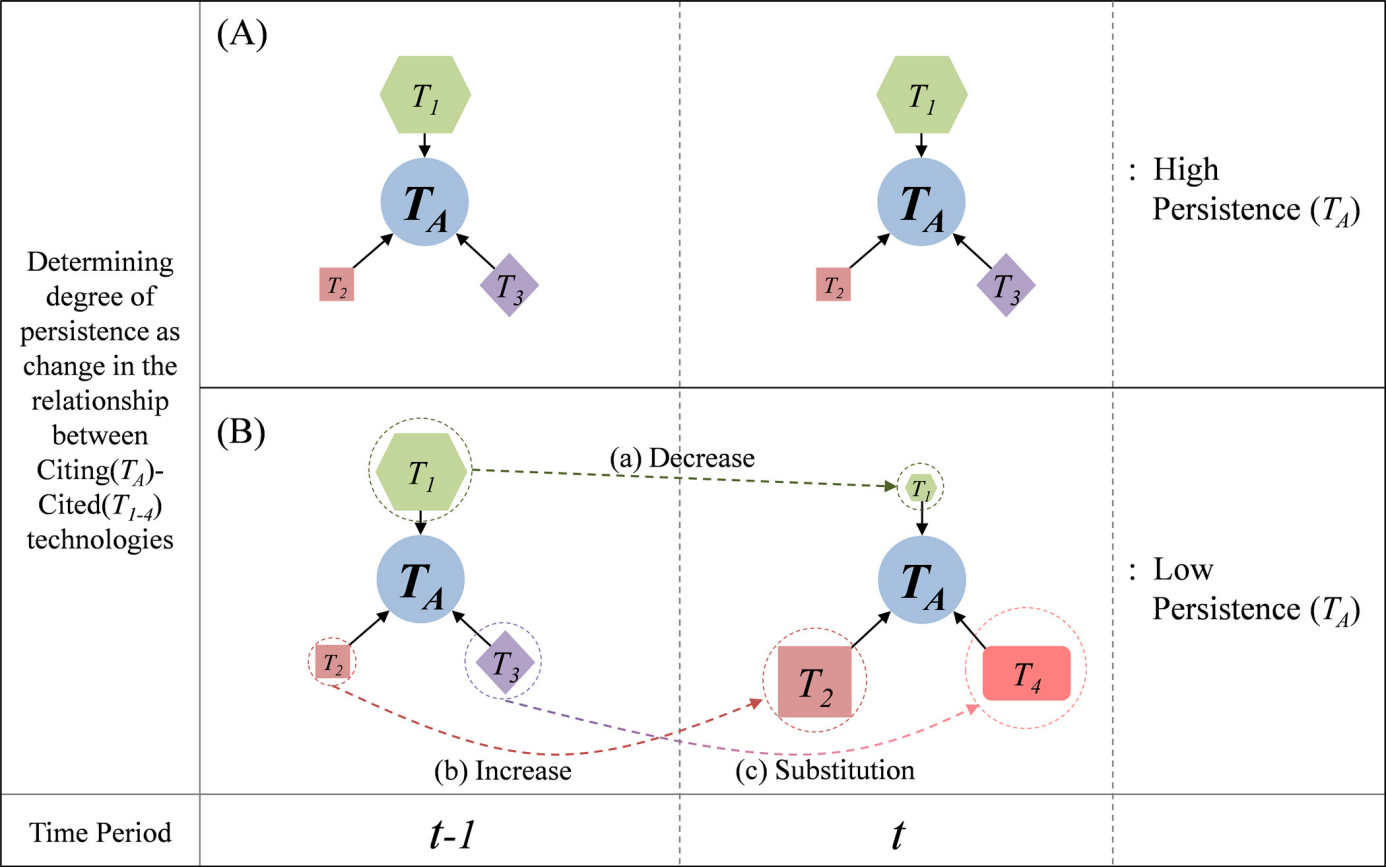
Conceptual Diagram for Determining Degree of Persistence.

The conceptual diagram for determining degree of persistence is illustrated in Fig 4.

Fig 4 shows how we determine the degree of persistence of Technology *A* (*T*_*A*_). Suppose that *T*_*A*_ cited Technologies *1*, *2*, and *3* (*T*_*1-3*_) at period *t-1*. If *T*_*A*_ cited the same technologies (*T*_*1-3*_) at the same level at period *t*, then *T*_*A*_ is characterized by high persistence, as shown in Fig 4−(A). On the other hand, if *T*_*A*_ cited *T*_*1*_ less (Fig 4−(B)−(a)), *T*_*2*_ more (Fig 4−(B)−(b)), *T*_*4*_ newly, and did not cite *T*_*3*_ (Fig 4−(B)−(c)) at period *t*, *T*_*A*_ is characterized by low persistence.

## Results

### Comparison between indicators

The correlations are analyzed to quantify the dependence of the measures of diversity and persistence. The values of the correlations between these measures are presented in Table 7.

**Table 7:** Pearson Correlations between Diversity and Persistence Measures.

**1991–1995**	**Diversity**	**Persistence**	**1996–2000**	**Diversity**	**Persistence**
**Diversity**	1.00	**0.46**	**Diversity**	1.00	**0.38**
**Persistence**	-	1.00	**Persistence**	-	1.00
**2001–2005**	**Diversity**	**Persistence**	**2006–2010**	**Diversity**	**Persistence**
**Diversity**	1.00	**0.48**	**Diversity**	1.00	**0.39**
**Persistence**	-	1.00	**Persistence**	-	1.00

According to the results, the value of correlation coefficients are in a range of 0.38−0.48 which implies that the indicators between diversity and persistence were not highly correlated with one another [69]. Furthermore, we undertook a comparative analysis of the correlations between other well-established diversity indexes, such as the variety, Shannon index, and Simpson index, and the two indicators (Rao-Stirling diversity and persistence), which can be found in S1 File.

### Dynamic convergence patterns in the ICT, BT, and NT sectors in the US

The results of the comparative analysis for the three main sectors and three subcategories of ICT for each period, as well as the diversity and persistence values, are shown in Tables 8 and 9, respectively. Based on our results, we discuss the main features of each of the three technology sectors in the US.

**Table 8:** Diversity and Persistence in ICT, BT, and NT Sectors in the US.

**Ranking**	**Diversity**							
	**1991–1995**		**1996–2000**		**2001–2005**		**2006–2010**	
	**Sector**	**Value**	**Sector**	**Value**	**Sector**	**Value**	**Sector**	**Value**
1	CE	0.3160	COM	0.3239	NT	0.3843	NT	0.4003
2	COM	0.3135	CE	0.3231	COM	0.3376	COM	0.3344
3	ICT	0.3077	ICT	0.3139	CE	0.3306	ICT	0.3233
4	BT	0.2950	NT	0.3135	ICT	0.3256	CE	0.3231
5	TEL	0.2937	TEL	0.2947	TEL	0.3087	TEL	0.3122
6	NT	0.2584	BT	0.2813	BT	0.2793	BT	0.2721
**Ranking**	**Persistence**							
	**1991–1995**		**1996–2000**		**2001–2005**		**2006–2010**	
	**Sector**	**Value**	**Sector**	**Value**	**Sector**	**Value**	**Sector**	**Value**
1	COM	0.9642	COM	0.9858	COM	0.9688	TEL	0.9645
2	ICT	0.9584	BT	0.9628	TEL	0.9510	BT	0.9499
3	TEL	0.9561	ICT	0.9538	BT	0.9478	COM	0.8976
4	CE	0.9548	TEL	0.9535	ICT	0.9296	ICT	0.8968
5	BT	0.8611	CE	0.9220	NT	0.8959	NT	0.8802
6	NT	0.7337	NT	0.7208	CE	0.8690	CE	0.8283

**Table 9:** Diversity and Persistence Values of IPCs in ICT, BT, and NT Sectors in the US.

Sector	Characteristics	Rank	1991–1995		1996–2000		2001–2005		2006–2010	
			IPC	Value	IPC	Value	IPC	Value	IPC	Value
**ICT**	**Diversity**	1	H04R	0.3837	B41K	0.3831	B07C	0.3845	B07C	0.4208
		2	H03J	0.3768	B07C	0.3808	H03H	0.3830	H01P	0.4012
		3	B07C	0.3751	H03H	0.3758	H04R	0.3788	G02F	0.3831
		4	H03H	0.3621	H03J	0.3712	B41K	0.3773	H04R	0.3786
		5	B41K	0.3606	H04R	0.3701	G09C	0.3734	H03G	0.3757
	**Persistence**	1	H03K	0.9993	G11C	0.9992	H01Q	0.9995	G11C	0.9985
		2	G03G	0.9987	G11B	0.9991	G11B	0.9991	G11B	0.9980
		3	G11C	0.9984	G03G	0.9990	G03G	0.9977	H03F	0.9979
		4	H04N	0.9983	H01S	0.9988	G01S	0.9977	H01S	0.9978
		5	G10L	0.9981	G10L	0.9981	H03F	0.9958	G03G	0.9971
**Telecommunications**	**Diversity**	1	H03H	0.3621	H03H	0.3758	H03H	0.3830	H01P	0.4012
		2	G09C	0.3577	H03B	0.3456	G09C	0.3734	H03H	0.3718
		3	G08C	0.3434	G08C	0.3454	H01P	0.3664	G09C	0.3530
		4	H03B	0.3299	H01P	0.3383	G08C	0.3376	H01Q	0.3508
		5	H01P	0.3169	G09C	0.3314	H03B	0.3353	H01S	0.3416
	**Persistence**	1	H01S	0.9977	H01S	0.9988	H01Q	0.9995	H01S	0.9978
		2	H04M	0.9975	H01Q	0.9979	G01S	0.9977	G01S	0.9967
		3	G01S	0.9975	H01P	0.9966	H04B	0.9952	H04B	0.9931
		4	H01Q	0.9973	H04M	0.9955	H01P	0.9929	H01Q	0.9927
		5	H01P	0.9942	H03D	0.9907	H04M	0.9888	H04M	0.9857
**Consumer electronics**	**Diversity**	1	H04R	0.3837	H03J	0.3712	H04R	0.3788	H04R	0.3786
		2	H03J	0.3768	H04R	0.3701	H03J	0.3721	H03G	0.3757
		3	H03G	0.3450	H03G	0.3451	H03G	0.3533	H03J	0.3632
		4	H03F	0.3442	H04S	0.3258	H04S	0.3396	H03F	0.3460
		5	H04H	0.2821	H03F	0.3131	H04H	0.3248	H04H	0.3087
	**Persistence**	1	H04N	0.9983	G11B	0.9991	G11B	0.9991	G11B	0.9980
		2	G11B	0.9978	H04N	0.9959	H03F	0.9958	H03F	0.9979
		3	H03F	0.9946	H03F	0.9904	H04N	0.9918	H04R	0.9951
		4	H04R	0.9757	H03G	0.9851	H04R	0.9786	H04N	0.9874
		5	H04H	0.9666	H04R	0.9837	H03G	0.9591	H03G	0.9605
**Computers and office machinery**	**Diversity**	1	B07C	0.3751	B41K	0.3831	B07C	0.3845	B07C	0.4208
		2	B41K	0.3606	B07C	0.3808	B41K	0.3773	G02F	0.3831
		3	G09G	0.3563	G09G	0.3594	G02F	0.3721	G09G	0.3652
		4	G05F	0.3476	G05F	0.3444	G09G	0.3617	B41J	0.3635
		5	G02F	0.3426	G02F	0.3419	G05F	0.3612	G05F	0.3618
	**Persistence**	1	H03K	0.9993	G11C	0.9992	G03G	0.9977	G11C	0.9985
		2	G03G	0.9987	G03G	0.9990	H03K	0.9924	G03G	0.9971
		3	G11C	0.9984	G10L	0.9981	G05F	0.9917	G05F	0.9938
		4	G10L	0.9981	H03K	0.9958	G11C	0.9906	H03L	0.9921
		5	H03L	0.9927	G02F	0.9938	H03L	0.9838	B41J	0.9899
**BT**	**Diversity**	1	C12S	0.3628	C12S	0.3715	C12S	0.3741	C12S	0.3878
		2	C02F	0.3517	C02F	0.3513	C02F	0.3633	C02F	0.3738
		3	G01N	0.3296	G01N	0.3390	C07G	0.3583	G01N	0.3675
		4	C12M	0.3260	C12M	0.3362	G01N	0.3552	C12M	0.3060
		5	C07G	0.3253	C07G	0.3230	C12M	0.3108	C07G	0.2610
	**Persistence**	1	G01N	0.9991	C02F	0.9979	A61K	0.9981	G01N	0.9985
		2	A61K	0.9959	G01N	0.9975	A01H	0.9954	C07K	0.9984
		3	C02F	0.9915	A61K	0.9963	C12N	0.9929	C12Q	0.9981
		4	C12Q	0.9873	C12N	0.9847	G01N	0.9926	A01H	0.9963
		5	C12M	0.9843	C12M	0.9846	C12Q	0.9924	A61K	0.9940
**NT**	**Diversity**	1	C01G	0.3762	C01G	0.3935	B81B	0.4251	B81B	0.4398
		2	C03C	0.3597	B01J	0.3835	C03C	0.4026	B82B	0.4195
		3	B01J	0.3453	C01B	0.3774	B82B	0.4003	C03C	0.4100
		4	C01B	0.3359	C03C	0.3688	C01B	0.3940	C01G	0.4034
		5	C23C	0.3290	C23C	0.3370	C01G	0.3846	C03B	0.4013
	**Persistence**	1	C23C	0.9954	C03B	0.9957	C23C	0.9889	C01B	0.9873
		2	C03B	0.9953	C01B	0.9895	C03B	0.9806	C03B	0.9692
		3	C01B	0.9948	C23C	0.9895	C01G	0.9786	C23C	0.9660
		4	B01J	0.9900	C03C	0.9839	B01J	0.9755	B01J	0.9651
		5	C01G	0.9663	B01J	0.9682	C01B	0.9731	C03C	0.9142

The top five IPCs of diversity and persistence for each technology sector are shown here. See S2 File for complete information.

#### ICT sector

The most active convergent movements in the ICT sector occurred during the early 1990s, when heterogeneous technologies (diversity) and consistent utilization of integrated technologies (persistence) were sought. Thereafter, this sector constantly remained at the middle level of persistence and diversity, when compared to the BT and NT sectors. From the aspect of the subsectors of ICT, computers and office machinery (COM) maintained the highest degree of persistence during 1991–2005; after the mid-2000s, telecommunications (TEL) held this position. From the viewpoint of diversity, consumer electronics (CE) showed the greatest push for adopting different technologies before the 2000s, replaced by the COM subsectors in the 2000s. In terms of class, both the H03K (Pulse technique) and G11C (Static stores) classes in the COM subsectors enjoyed the highest degree of persistence during 1991–2010, with the exception of 2001–2005, when the TEL subsectors H01Q (Aerials) held top position. By contrast, since the mid-1990s, the B07C (Postal sorting) class from the COM subsector has been the fastest growing class to absorb different technology fields.

The abovementioned changes may be explained by some historical events. In the early 1990s, important barriers (e.g., the Advanced Research Projects Agency Network and National Science Foundation Network) were decommissioned, thereby permitting commercial traffic on the internet. This had a revolutionary impact on both culture and commerce. A series of government efforts, such as the National Information Infrastructure Act of 1991 and Telecommunication Act of 1996, were introduced to accelerate commercialization via the Internet [70]. Consequently, tremendous technological efforts occurred in the development of communication equipment, computer networks, and cellular phone networks to support the new digital economy. This can be interpreted as telecommunications in the US being able to retain technological competence, while expanding in other areas, such as online commerce. By contrast, the development speed of personal computers (PCs), the core component of the ICT sector, and shortened life cycles, responding to demand changes of software and rapid changes in computer-related technology, led to a drop in profitability and discouragement of the development of PC-related technologies [71]. The digital satellite dish, digital versatile disc, and digital music player were all successfully launched as consumer electronics from the mid-1990s to the mid-2000s. During that time, the consumer electronics industry made an effort to provide more useful devices to consumers by combining telecommunications with computer technologies [72].

#### BT sector

Compared with the ICT and NT sectors, BT has shown a high propensity for consistently utilizing integrated technologies (persistence) and low propensity for absorbing heterogeneous technologies (diversity) since the mid-1990s. In detail, the degree of persistence for the classes of G01N (Investigating or analyzing materials by determining their chemical or physical properties), A61K (Preparations for medical, dental, or toilet purposes), and C02F (Treatment of water, wastewater, sewage, or sludge) remained at the highest position in the 1990s. Meanwhile, the classes of A01H (New plants or processes for obtaining them) and C07K (Peptides) appeared to have built their technological competences in the 2000s. From the diversity perspective, C12S (Processes using enzymes or microorganisms to liberate, separate, or purify a pre-existing compound or composition) and C02F (Treatment of water, wastewater, sewage, or sludge) have played key roles in constantly accepting other technologies. These distinctive features of the BT sector can reflect one of its predominant features: firms strive to develop various vertically integrated functions to prove that their technologies are viable in high-risk business environments [73]. In addition, The Human Genome project, conducted for 13 years (1990–2003) to identify the chemical base pairs present in human DNA for use in further biological studies, contributed a revolution in BT innovation, and played a key role in making the US the global leader [74].

#### NT sector

Compared to the other two sectors, the NT sector has actively expanded the scope of its various applications (diversity). In particular, the classes of C01G (Compounds containing metals) and B81B (Micro-structural devices or system) played a vital role in such trends during the 1990s and 2000s, respectively. Moreover, NT has an intrinsic characteristic whereby the availability of nanostructures with highly controlled properties has expanded significantly [75]. Thus, it includes the production and application of physical, chemical, and biological systems at the nanoscale, as well as the integration of the resulting nanostructures into larger systems [76]. This distinguished feature of NT was reflected in the results of this study.

From a historical perspective, a number of important technological breakthroughs or completions, such as the carbon nanotube, thin film, and atomic force microscopy, occurred in the late 1980s and early 1990s. These developments influenced the further development of NT [77]. The National Nanotechnology Initiative program, launched in 2000, became a trigger for intensive research, making important contributions to national goals in a variety of sectors, such as health, information technology, and manufacturing [78].

### Dynamic convergence patterns in ICT, BT, and NT sectors in Korea

The results of the comparative analysis for the three main sectors and three subcategories of ICT for each period, as well as the diversity and persistence values, are shown in Tables 10 and 11, respectively. Based on our results, we discuss the main features of each of the three technology sectors in Korea.

**Table 10:** Diversity and Persistence in ICT, BT, and NT Sectors in Korea.

**Ranking**	**Diversity**							
	**1991–1995**		**1996–2000**		**2001–2005**		**2006–2010**	
	**Sector**	**Value**	**Sector**	**Value**	**Sector**	**Value**	**Sector**	**Value**
1	COM	0.2707	COM	0.2539	NT	0.2805	NT	0.3566
2	TEL	0.2674	TEL	0.2469	TEL	0.2561	TEL	0.2667
3	NT	0.2476	ICT	0.2458	ICT	0.2435	ICT	0.2582
4	ICT	0.2463	CE	0.2367	CE	0.2397	CE	0.2552
5	CE	0.2008	NT	0.2293	COM	0.2348	COM	0.2527
6	BT	0.1474	BT	0.1866	BT	0.2246	BT	0.2181
**Ranking**	**Persistence**							
	**1991–1995**		**1996–2000**		**2001–2005**		**2006–2010**	
	**Sector**	**Value**	**Sector**	**Value**	**Sector**	**Value**	**Sector**	**Value**
1	COM	0.6564	TEL	0.8225	COM	0.8665	TEL	0.9023
2	ICT	0.5392	COM	0.7856	TEL	0.8519	COM	0.8694
3	TEL	0.4953	ICT	0.7615	ICT	0.7966	ICT	0.8340
4	CE	0.4661	CE	0.6764	NT	0.6756	BT	0.7341
5	NT	0.2230	BT	0.5016	CE	0.6716	CE	0.7302
6	BT	0.0905	NT	0.4760	BT	0.6602	NT	0.6557

**Table 11:** Diversity and Persistence Values of IPCs in ICT, BT, and NT Sectors in Korea.

Sector	Characteristics	Rank	1991–1995		1996–2000		2001–2005		2006–2010	
			IPC	Value	IPC	Value	IPC	Value	IPC	Value
**ICT**	**Diversity**	1	H01P	0.3568	G01S	0.3911	H04R	0.3772	H04R	0.3742
		2	H01Q	0.3565	H04R	0.3689	H03H	0.3732	B07C	0.3573
		3	G01S	0.3546	H03H	0.3613	G01S	0.3196	H03H	0.3379
		4	H03H	0.3494	B41J	0.3347	H03G	0.3192	H03G	0.3231
		5	G10L	0.3455	H03L	0.3243	G05F	0.3186	G01S	0.3142
	**Persistence**	1	H04N	0.9969	H04N	0.9977	G11C	0.9991	G11C	0.9994
		2	G11C	0.9937	G11B	0.9942	G03G	0.9988	G03G	0.9993
		3	G11B	0.9926	H03K	0.9933	G11B	0.9985	G02F	0.9988
		4	H03K	0.9837	H04M	0.9887	H04N	0.9970	H01Q	0.9982
		5	G03G	0.9733	B41J	0.9880	H01Q	0.9963	G10L	0.9956
**Telecommunications**	**Diversity**	1	H01P	0.3568	G01S	0.3911	H03H	0.3732	H03H	0.3379
		2	H01Q	0.3565	H03H	0.3613	G01S	0.3196	G01S	0.3142
		3	G01S	0.3546	H01S	0.2950	H03D	0.2954	H01P	0.3060
		4	H03H	0.3494	H03D	0.2892	H01S	0.2923	H01S	0.3035
		5	H03B	0.3053	G08C	0.2810	H03M	0.2753	H03M	0.3014
	**Persistence**	1	H04M	0.9502	H04M	0.9887	H01Q	0.9963	H01Q	0.9982
		2	H01S	0.9096	H01S	0.9595	H04L	0.9947	H04Q	0.9931
		3	H04B	0.8787	H03M	0.9440	H01S	0.9935	H01S	0.9931
		4	H01Q	0.8175	H01Q	0.9383	H03M	0.9896	H01P	0.9903
		5	H04L	0.7593	H04B	0.9357	H01P	0.9833	H04L	0.9889
**Consumer electronics**	**Diversity**	1	H04R	0.3320	H04R	0.3689	H04R	0.3772	H04R	0.3742
		2	H03F	0.2856	H03G	0.2979	H03G	0.3192	H03G	0.3231
		3	H03G	0.2822	H04S	0.2870	H03F	0.3106	H03J	0.2857
		4	H04H	0.2699	H04H	0.2645	H04N	0.2496	H04H	0.2831
		5	G11B	0.2428	H04N	0.2356	H04H	0.2473	H04N	0.2753
	**Persistence**	1	H04N	0.9969	H04N	0.9977	G11B	0.9985	H04N	0.9946
		2	G11B	0.9926	G11B	0.9942	H04N	0.9970	G11B	0.9942
		3	H03G	0.9048	H03G	0.9109	H03F	0.9839	H03F	0.9881
		4	H04R	0.5341	H03F	0.8991	H03G	0.9515	H04R	0.9728
		5	H03F	0.3003	H04R	0.8334	H04R	0.8822	H03G	0.9518
**Computers and office machinery**	**Diversity**	1	G10L	0.3455	B41J	0.3347	G05F	0.3186	B07C	0.3573
		2	G05F	0.3298	H03L	0.3243	G09G	0.3066	G10L	0.3106
		3	B07C	0.3273	G09G	0.3204	G10L	0.3019	H03K	0.3082
		4	G09G	0.3253	G02F	0.3195	H03K	0.2929	B41J	0.3046
		5	B41J	0.3169	G05F	0.3047	B41J	0.2860	G05F	0.3004
	**Persistence**	1	G11C	0.9937	H03K	0.9933	G11C	0.9991	G11C	0.9994
		2	H03K	0.9837	B41J	0.9880	G03G	0.9988	G03G	0.9993
		3	G03G	0.9733	G03G	0.9860	H03K	0.9953	G02F	0.9988
		4	G02F	0.8896	G11C	0.9853	G02F	0.9835	G10L	0.9956
		5	B41J	0.8837	G02F	0.9638	B41J	0.9828	H03K	0.9946
**BT**	**Diversity**	1	G01N	0.3698	C12M	0.3951	G01N	0.4003	G01N	0.3813
		2	C12M	0.3327	G01N	0.3533	C02F	0.3240	C02F	0.3273
		3	C02F	0.2688	C02F	0.3165	C12M	0.2906	C12M	0.3028
		4	A61K	0.1726	C07K	0.1937	C12Q	0.2801	A01H	0.2820
		5	C12P	0.1657	A61K	0.1886	A61K	0.2574	C12Q	0.2447
	**Persistence**	1	G01N	0.4982	A61K	0.9818	C12N	0.9822	C12N	0.9692
		2	A61K	0.4967	C12P	0.9578	C07K	0.9535	C07K	0.9638
		3	A01H	0.0000	G01N	0.9485	C12P	0.9477	A61K	0.9506
		4	C02F	0.0000	C12N	0.9373	G01N	0.9401	C12Q	0.9495
		5	C07G	0.0000	C02F	0.9347	A61K	0.9371	G01N	0.9467
**NT**	**Diversity**	1	C03B	0.4078	C03B	0.3602	C01B	0.3689	B82B	0.3946
		2	C01B	0.3695	C03C	0.3454	B81B	0.3685	C03C	0.3823
		3	B01J	0.3245	C01G	0.3088	C03C	0.3652	C01G	0.3814
		4	C01G	0.3018	B01J	0.2827	C01G	0.3116	C03B	0.3695
		5	C03C	0.2942	C23C	0.2781	C03B	0.3060	C01B	0.3590
	**Persistence**	1	C23C	0.9161	C23C	0.9797	C23C	0.9925	C23C	0.9761
		2	C01B	0.3771	B01J	0.6905	C01G	0.9482	C03B	0.9592
		3	C01G	0.2572	C03C	0.6622	C03B	0.9370	C03C	0.9108
		4	B01J	0.2338	C03B	0.5379	C03C	0.9298	C01G	0.8805
		5	B81B	0.0000	C01B	0.4780	B01J	0.8995	B01J	0.7450

The top five IPCs of diversity and persistence for each technology sector are shown here. See the S2 File for complete information.

#### ICT sector

Compared to the NT and BT sectors, ICT has increased its level of persistence, while also maintaining the highest ranking of all three sectors during the period of analysis. In particular, the COM and TEL subsectors have played a leading role in the trend, more so than the CE subsector. For example, the H04M (Telephonic communication) and H01Q (Aerials) in the TEL subsector ranked at an all-time high during 1991–2000 and 2001–2010, respectively. In addition, the classes of H03K (Pulse technique) and G11C (Static stores) in the COM subsector achieved similar results. From the viewpoint of diversity, even though the NT sector took the lead during the 2000s, the COM and TEL subsectors absorbed other technologies at a very high rate during the 1990s and 2000s, respectively.

In the 1990s, the prevalence of PCs continuously increased and the availability of a high-speed communication network spread rapidly nationwide, allowing for the development of ICT-related components and internet-based ways of life. As the demand for mobile phone services increased, the Korean government designated a digital-based code division multiple access system as the standard, and successfully commercialized the world’s first mobile phone services in 1996 [79]. After building on the well-developed infrastructure, Korea reinforced its ICT competence with a series of polices, such as e-Korea vision 2006 in 2002, Broadband IT Korea 2007 in 2003, and the u-Korea plan in 2006, and high-quality and fast broadcasting/internet/communication services, such as the world’s first wireless broadband internet), world’s first high-speed downlink packet access commercial service in 2006, and real-time internet protocol television broadcasting in 2008 [80]. Consequently, on the basis of the ICT Development Index established by the International Telecommunication Union, Korea became one of the leading IT nations among 152 countries in 2011, followed by Sweden, Iceland, Denmark, and Finland (the US ranked 17^th^) [51].

#### BT sector

Compared to the ICT and NT sectors, BT was characterized by the lowest degree of diversity during the period of analysis. However, the result showed the highest growth in the degree of persistence (0.09 → 0.50: 455%) between the 1991–1995 and 1996–2010 time periods, followed by the NT (0.22 → 0.48: 113%) and ICT (0.54 → 0.76: 41%) sectors. Specifically, the classes of A61K (Preparations for medical, dental, or toilet purposes) and C12N (Microorganisms or enzymes) contributed to building the greatest stock of technological knowledge during 1991–2000 and 2001–2010, respectively. Although the BT sector was characterized by low degree of diversity, the classes of G01N (Investigating or analyzing materials by determining their chemical or physical properties), C12M (Apparatus for enzymology or microbiology), and C02F (Treatment of water, wastewater, sewage, or sludge) appeared to use heterogeneous technologies actively. This finding shows that the convergence capability of Korea in the BT sector considerably caught up with that of the US at the analytic basis of class; however, the C12S (Processes using enzymes or microorganisms to liberate, separate, or purify a pre-existing compound or composition) class, which ranked at the top for the entire period in the US, did not improve in Korea.

Historically the BT sector had a relatively shorter experience in Korea than did the ICT sector [51]. In the 1990s, the Korean BT sector in its nascent stage attempted to improve its technological foundations. As such, in 1994, the government launched the basic plan for the promotion of BT, Biotech 2000 (1994–2006), to accomplish its goals with a budget of Won 15.5 trillion (US$ 18 billion) [81]. In 2006, the second framework plan for pan-governmental BT promotion, BioVision 2016 (2007–2016), was established to transform BT into a national economic growth sector, with a budget of Won 14.3 trillion (US$ 16.6 billion) [52]. This result is consistent with the finding that these strong efforts of the Korean government helped increase the innovation capacity in BT [82–84].

#### NT sector

The NT sector was distinguished by intensively diversifying technological applications in the 2000s. Specifically, the classes of C03B (Compounds containing metals), C01B (Non-metallic elements), and B81B (Micro-structural devices or system) contributed to the increase in the degree of diversity during the 1990s and 2000s, respectively. Meanwhile, as mentioned, this sector dramatically enhanced the level of persistence, following the BT sector. In particular, the C23C (Coating metallic material) class contributed to this technological improvement most significantly. In the meantime, the C03B (Manufacture, shaping, or supplementary processes) class accomplished the most advancement among the other classes. From the early 1990s, a number of government-funded research projects, such as intelligent microsystems, terra level nanodevices, and nano-structured material technologies, were begun initially because of the risk and uncertainty of NT [84]. The Korean government buckled down to invest in this sector with the launch of the Korea National Nanotechnology Initiatives in 2001, which supported the required legal and institutional backing for the industry over a 10-year period with $US 1.4 billion. Such strong government stimulation brought not only considerable improvement in the accumulated knowledge in NT, but also encouraged the expansion of other applications, such as semiconductors, automobiles, secondary batteries, and displays, in the 2000s [54].

### Comparison between US and Korea

In spite of the substantial differences across countries, from economic sizes to regions, many studies [85–87] have shown that it is reasonable to compare nations from the technology perspective. In this study, we have taken into account two main dimensions of technological convergence that play a comparable role in the making of the capabilities for technology convergence of a nation. Thus, the degrees of diversity and persistence laid the foundation for the key factors influencing national competitiveness.

In order to properly compare the levels of capabilities for technology convergence between the US and Korea, we normalized all values into the range of [0, 1], as shown in Table 12.

**Table 12:** Diversity and Persistence Values and Normalized Values in ICT, BT, and NT Sectors between the US and Korea.

			Value				Normalized Value (Min: 0 / Max: 1)			
Measurement	Technology Sector	Country	1991–1995	1996–2000	2001–2005	2006–2010	1991–1995	1996–2000	2001–2005	2006–2010
**Diversity**	**ICT**	**US**	0.3077	0.3139	0.3256	0.3233	0.6338	0.6582	0.7046	0.6952
		**KR**	0.2463	0.2458	0.2435	0.2582	0.3910	0.3891	0.3800	0.4379
	**BT**	**US**	0.2950	0.2813	0.2793	0.2721	0.5835	0.5294	0.5212	0.4929
		**KR**	0.1474	0.1866	0.2246	0.2181	0.0000	0.1548	0.3052	0.2796
	**NT**	**US**	0.2584	0.3135	0.3843	0.4003	0.4390	0.6567	0.9368	1.0000
		**KR**	0.2476	0.2293	0.2805	0.3566	0.3960	0.3238	0.5262	0.8272
**Persistence**	**ICT**	**US**	0.9584	0.9538	0.9296	0.8968	0.9949	0.9896	0.9619	0.9243
		**KR**	0.5392	0.7615	0.7966	0.8340	0.5144	0.7692	0.8095	0.8523
	**BT**	**US**	0.8611	0.9628	0.9478	0.9499	0.8834	1.0000	0.9827	0.9851
		**KR**	0.0905	0.5016	0.6602	0.7341	0.0000	0.4713	0.6531	0.7378
	**NT**	**US**	0.7337	0.7208	0.8959	0.8802	0.7374	0.7226	0.9233	0.9053
		**KR**	0.2230	0.4760	0.6756	0.6557	0.1520	0.4419	0.6707	0.6480

**Fig 5:**
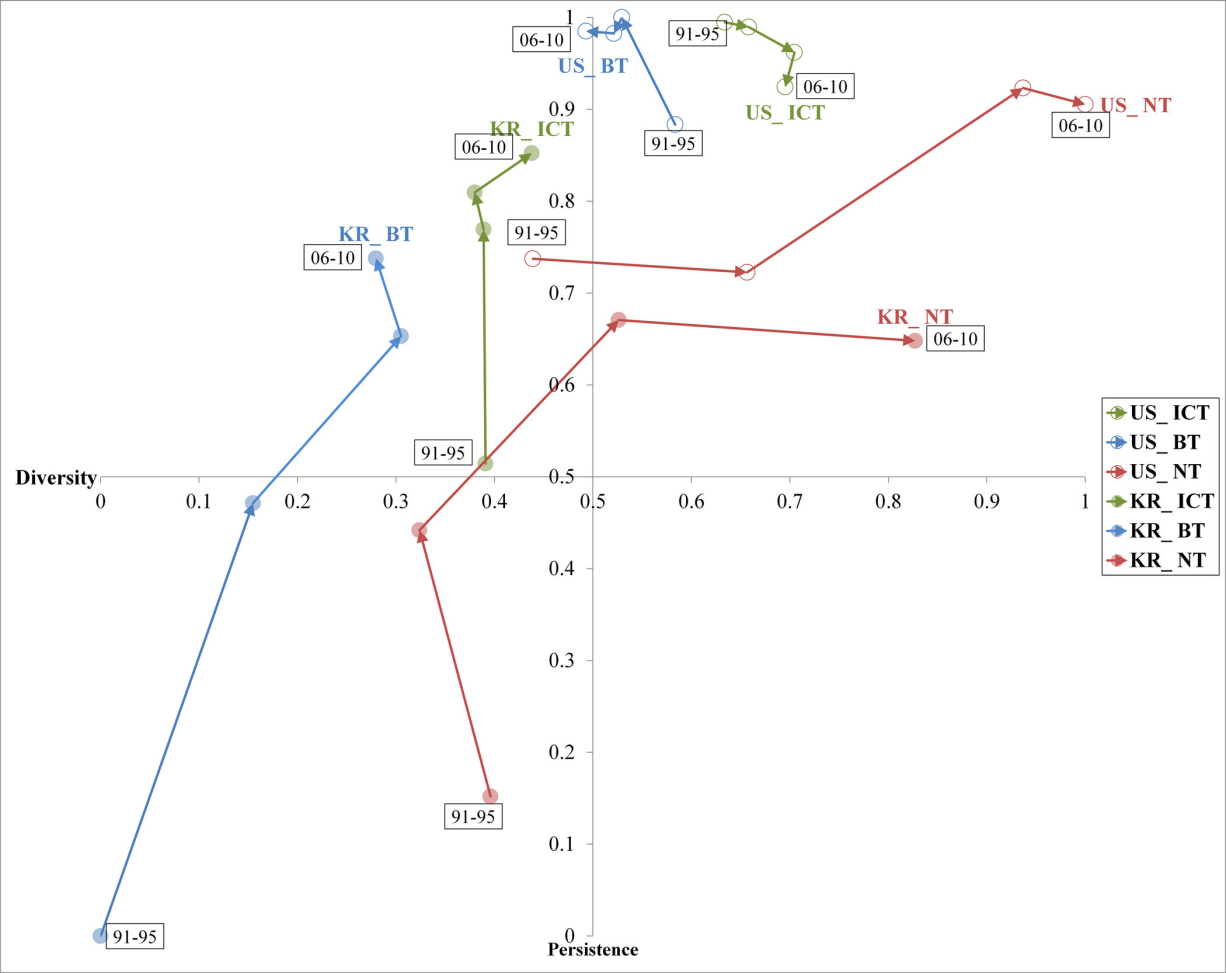
Normalized Diversity and Persistence Patterns in the US and Korea by Time Period. Diversity value range: 0–1; Persistence value range: 0–1. Quadrant 1: Sustainability zone; Quadrant 2: Specialization zone; Quadrant 3: Improvement zone; Quadrant 4: Diversification zone.

Fig 5 represents the three main sectors in the US and Korea per time period, as shown in Tables 8 and 10, and shows how dynamic patterns of the three technology sectors for both countries have evolved in the process of technological convergence, when the normalized diversity value is set as the horizontal line (X axis) and the normalized persistence value as the vertical line (Y axis).

**Fig 6:**
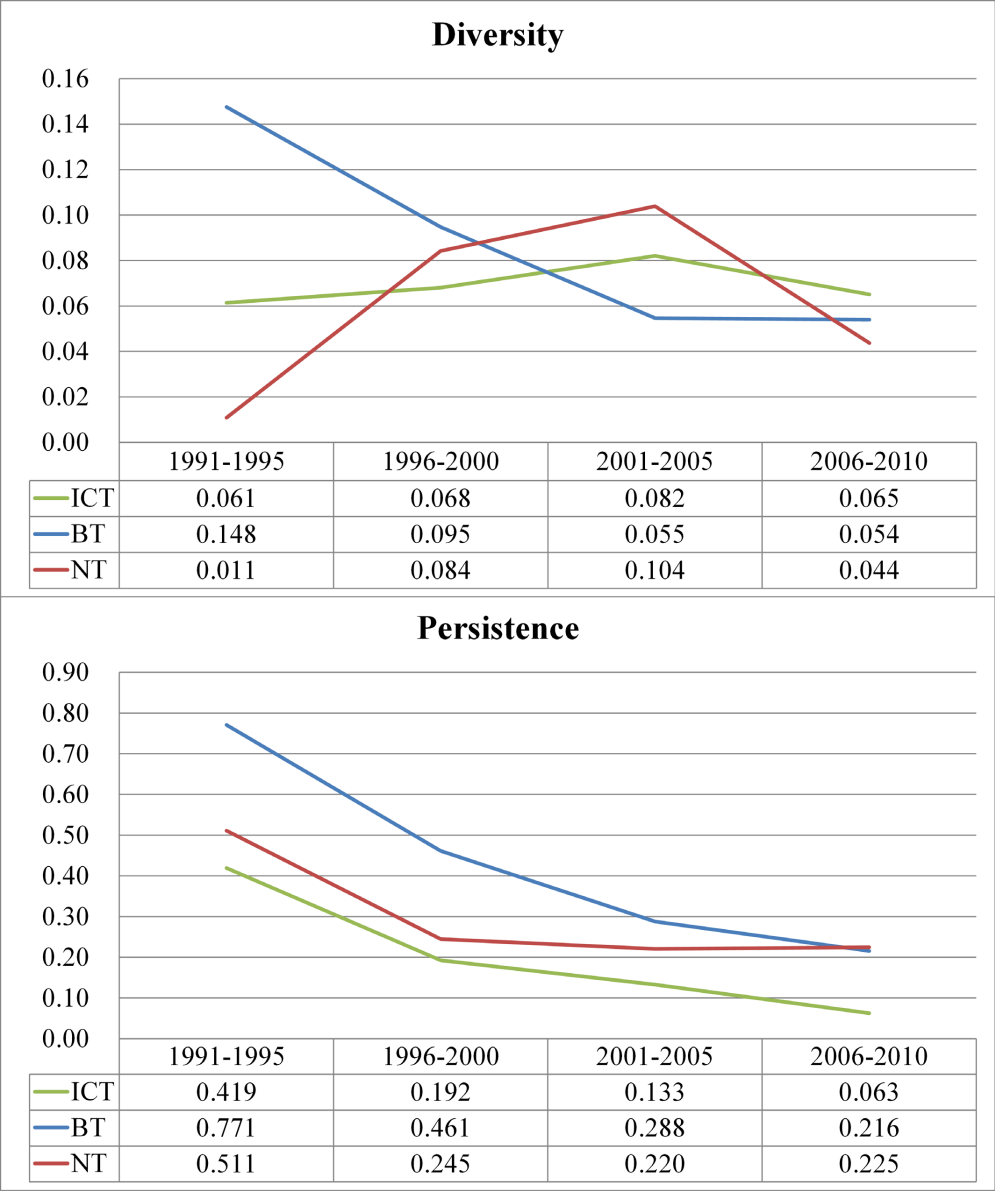
Pattern of Difference in Technology Convergence in ICT, BT, and NT Sectors between the US and Korea.

As Brusoni and Geuna [38] stressed, persistent efforts to absorb and adopt technologies over time lead to increasing specialization, and for the US, the ICT sector has experienced a declining degree of specialization. Meanwhile, the BT sector has improved its technological specialization. Conversely, the two sectors showed opposite directions from the diversity perspective. The NT sector considerably reinforced both the level of persistence and diversity. In the case of Korea, the degree of technology convergence in those sectors has improved dramatically since the 1990s. Specifically, the ICT and BT sectors have tended to concentrate on solidifying their core technological specialization more than pursuing the application of different territories. Meanwhile, like the US, the development direction of Korea’s NT sector was similarly increasing, although there was a difference in the order of priorities in terms of diversity and persistence. To put it precisely, we examined the changes in the gap of the level of technology convergence over time in terms of diversity and persistence, as shown in Fig 6.

From the diversity perspective, the differentials in the capabilities of convergence for each technology sector between the US and Korea presents irregular trends. In detail, from the standpoint of Korea, the discrepancy in the capability for diversity in the BT sector dropped sharply until the mid-2000s and then was maintained, while the distance in the NT sector continuously widened until the mid-2000s and then decreased dramatically. By contrast, the differential in the ICT sector has maintained subtle fluctuations. On the contrary, the gaps of capabilities that related to persistence in the three sectors have decreased consistently.

Our results were drawn from the relative comparison between countries, and thus, they should be taken with caution. However, the results suggest some interesting implications. First, Castellacci and Archibugi [85] and Archibugi and Coco [87] demonstrated that through rapid improvements in its technological capabilities in the 1990s, Korea moved toward the advanced technology club of nations, characterized by high levels of creation and diffusion of knowledge in 2000. Although the indicators of those studies, which consist of a set of indicators on various aspects of a nation’s technological capability, were incomparable to our indicators due to somewhat different perspectives, both their and our empirical results are comparable in terms of the changes in the degree of innovative capability of nations, which is an essential requirement for the modern knowledge-based economy to compete [87]. This is not to argue that one approach is better than another is, but rather to point out that our indicators can provide more useful insights in the study of the technological convergence process. Given that the capabilities of technological convergence have affected the competitiveness of nations since the 1990s, innovation scholars or policymakers should reconsider the role of the interaction between absorbing heterogeneous technologies and consistently using aggregated technologies.

Second, the resource-based view explains competitive advantage based on the premise that heterogeneity in resources and capabilities differentiates close competitors. Helfat and Peteraf [88] emphasized that it is necessary to identify patterns in the evolution of organizational capabilities over time to explain the sources that create competitive advantage. Thus, from the viewpoint of the resource-based view, our results provide empirical evidence on the changes of capabilities for technological convergence, which may be one of the prime components that create the competitive heterogeneity of a nation. According to our results, while the Korean government somehow has achieved its purpose during the last two decades, there are indications that there still exists a considerable gap of diversity compared to the US. This provides meaningful insight into the transition of science and technology policy that develops a system to coordinate these two activities, eventually completing a virtuous circle to enhance the capabilities of technological convergence of the nation.

## Discussion

Unlike prior studies, which suggested various analytical approaches with a lack of understanding on the phenomenon of technology convergence, this study has begun to propose a robust framework to measure this phenomenon. In order to accomplish this goal, we identified the distinctive properties of the convergence phenomenon by investigating a variety of theories’ core concepts and, then, suggested the proper terms and indicators reflected for them. Going through this procedure, the present study presents three important outcomes as follows.

First, we conceptualized the phenomenon of technology convergence in terms of diversity and persistence. In the process of convergence, diversity occurs to solve a problem beyond its domain, thereby improving the absorption capability across heterogeneous technology fields. Meanwhile, persistence creates specialization built by the continuous usage of accumulated knowledge over time. The interaction of the concepts of diversity and persistence provide a foundation for the development of an analytic framework in which to compare the differences and patterns of convergence competence by country at the technology sector level. It is significant that the suggested framework reflects the assertion by Kodama [89] that technology convergence is a course of integrating knowledge, which belongs to different areas of specialization in a broad sense. The framework has the potential to apply to any social study that is based on the idea that the source of organizational competence is derived from the integration of specialized knowledge [90]. Such studies may establish the external validity of the framework and indicators, gaining consensus on the nature and methodology for the phenomenon.

Second, we demonstrated how to operationalize the framework based on patent analysis at the country level, in areas of major technology convergence, such as the ICT, BT, and NT sectors. Moreover, we illustrated how these three technology sectors evolved and identified which area (e.g., IPC subclass) played a leading role in each sector in both the US and Korea during 1991–2010. Although it is acknowledged that the US has considerable technological competences compared to Korea, the result of the analysis explicitly verified the comparable patterns of the convergence process for the technology sectors of the two countries, and indicated the differences in the core technology fields for each nation. Our results indicate that Korea has narrowed its level of core capability in emerging high-tech sectors, whereas the capabilities for diversity in the ICT and BT sectors can be seen as a sign of relative weakness compared to the US. This evidence may present empirical evidence for the creation of differentiation of national competitiveness.

Finally, this study offers strategic directions for redistributing the appropriate combination of resources to enhance the capabilities for technology convergence. As described in the previous paragraph, the results allow us to observe the dynamic patterns of the convergence phenomenon and to indicate the difference of core capabilities behind the phenomenon. These implications enable us to ascertain the desirable and intended outcomes of government innovation policy, thereby enabling policymakers to reconsider the purpose of the current policy. For instance, Korea confronted the limitation of technology adoption from the strategy of advanced nations in the mid-1990s, after losing its competitive advantages from low labor costs [91]. The government has worked on establishing a long-term policy to acquire core capabilities in the ICT sector, which already possessed national competitiveness, after the mid-1990s; thereafter, a series of strong national promotion plans for the BT and NT sectors was launched to improve the depth of technological specialization in the early 2000s. According to this study, such government-driven support consequentially revealed the finding that all three sectors dramatically increased their technological improvements. However, compared to the US, there are still considerable gaps in the capabilities for persistence and diversity in the three sectors. Thus, it is necessary for Korea to establish a plan that may accomplish desired outcomes, which could increase the degree of capabilities equally for both persistence and diversity or concentrate one more than the other.

The limitations of this study present some challenging questions for future research. First, in order to understand the role of capabilities for technology convergence at a country level, it is necessary to make an effort to estimate countries’ capabilities by taking into consideration economic factors, such as economic level, labor cost, and demand [92], and/or other technological factors, such as human capital, technological infrastructure [86], and research and development (R&D) intensity [93]. In addition, it is an important task to investigate the role of capabilities for technological convergence in economic growth. This analysis may contribute to the literature on how the capabilities of technology convergence are associated with economic growth, and which factor has more influence. Second, an inherent limitation concerns the use of the patent dataset from the USPTO. Criscuolo [94] pointed out that many studies that use patents granted by the USPTO might underestimate the R&D activities of firms operating outside of the US. Furthermore, such studies might overestimate the R&D activities of US-based companies because of the home advantage effect. Therefore, the degree of convergence competence in the US may be exaggerated compared to that of other countries. Finally, an investigation is required into the circumstances under which certain events or actions caused different outcomes in an identical mechanism. In the beginning of this study, technological development, deregulation, and customers’ preferences were exemplified as significant drivers of this convergence. However, there are different socio-economic conditions among nations. Therefore, identifying the factors affecting the different outcomes would reinforce the usability of the framework.

## Supporting Information

S1 File Pearson Correlation between Diversity and Persistence. *N* = Variety of IPCs; *H* = Shannon index; *I* = Simpson index; *D* = Rao–Stirling index; *P* = Persistence. The highest correlations are shaded grey. The correlations between the different diversity and persistence measures are provided in S1 File. First, we compared the indicators and found that diversities *H*, *I*, and *D* were highly correlated. This result was consistent with the research of Rafols and Meyer [24]. The highest correlation was between *H* and *I* during the 1990s and between *H* and *D* during the 2000s.
(XLSX)

S2 File Diversity and Persistence Values for the US and Korea by Technology Sectors. (XLSX)

S3 File CitingIPC * CitedIPC * Period matrixes. This Excel file contains 15 matrixes. Rows are CitedIPCs and columns are CitingIPCs.
(XLSX)

S4 File Other information. This file contains: 1. patent count and proportion, 2. cosine distances between IPCs, 3. persistence value of IPCs and sectors, and 4. diversity value of IPCs and sectors.
(XLSX)

S5 FileExample of calculating diversity and persistence.The process for calculating the values of diversity and persistence of CitingIPC A01B in the US at the 1991–1995 time period has been demonstrated. This file contains seven sheets. The description for each sheet is as follows. A. Material_US_19861990_Raw_Matrix: the matrix is arranged in CitedIPC and CitingIPC in the US at the 1986–1990 time period to calculate the value of persistence. Yellow background color cells (B2:B639) are the element of vector A01B in the US at the 1986–1990 time period. B. Material_US_19911995_Raw_Matrix: the matrix is arranged in CitedIPC and CitingIPC in the US at the 1991–1995 time period to calculate the value of diversity and persistence. Yellow background color cells (B2:B639) are the element of vector A01B in the US at the 1991–1995 time period. C. Material_Wholetime_IPC Matrix: the matrix is arranged in CitedIPC and CitingIPC during the whole time period (1976–2010). A pair of column vectors is utilized to calculate distance between *i* and *j* (*d*_*ij*_) in Eq (2)$\left(d_{ij}=1-\frac{v_{i}∙v_{j}}{\left\|v_{i}\|\|v_{j}\right\|}\right)$. D. Diversity_Proportion(Pi, Pj): values in the yellow background color cells are used for calculating *p*_*i*_ and *p*_*j*_ in Eq (1) and are calculated by dividing each element (*i* or *j*) by the sum of all elements of vector A01B in the US at the 1991–1995 time period. Please refer to the Excel function of the D3:D640 cells. E. Diversity_Cosine Distance(Dij): values that indicate the distance between elements *i* and *j* (*d*_*ij*_) in Eq (2) are computed based on sheet C. Material_Wholetime_IPC Matrix. ***v***_***i***_ and ***v***_***j***_ in Eq (2) are a pair of column vectors in sheet C. Please refer to the Excel function in the B2 cell. F. Diversity_PiPjDij: value of diversity (0.721371) of CitingIPC A01B in the US at 1991–1995 time period is the sum of *p*_i_*p_j_*d_*ij*_ in the range of C3:XP640, where the value of *p*_*i*_ and *p*_*j*_ from sheet D are indicated in the range of B3:B640 and C2:XP2, respectively, and *d*_*ij*_ is the same as the value in the cells, matching elements *i* and *j* in sheet E. Please refer to the Excel function in the range of C3:XP640 and the A2 cell. G. Persistence: the persistence value (0.992882) of A01B in the US at the 1991–1995 time period is the cosine similarity between vectors of A01B at the 1986–1990 (C3:C640) and the 1991–1995 (D3:D640) time period. Refer to the Excel function of the E3 cell.(XLSX)
